# Differentiating autism spectrum disorder and global developmental delay in preschoolers: overlapping profiles and diagnostic challenges

**DOI:** 10.3389/fpsyg.2025.1690272

**Published:** 2025-11-14

**Authors:** Veronica Sperandini, Elisa Fucà, Martina Sbarbati, Maria Schettino, Stefania Falvo, Fabio Quarin, Paola De Rose, Stefano Vicari

**Affiliations:** 1 Child and Adolescent Neuropsychiatry Unit, Bambino Gesù Children's Hospital, IRCCS, Rome, Italy; 2 Child Neurology and Psychiatry Unit, Catholic University, Rome, Italy; 3 Department of Life Science and Public Health, Catholic University of the Sacred Heart, Rome, Italy

**Keywords:** global developmental delay, autism, differential diagnosis, adaptive functioning, preschool children

## Abstract

**Introduction:**

Differentiating Autism Spectrum Disorder (ASD) from Global Developmental Delay (GDD) in preschoolers may be challenging due to overlapping symptoms and shared developmental impairments. This study aimed to examine similarities and differences in adaptive functioning, emotional and behavioral characteristics, and autistic symptomatology in preschoolers with ASD and those with GDD presenting autistic traits.

**Methods:**

Eighty-nine children aged 3 to 5.8 years (42 with ASD, 47 with GDD), matched for age, intelligence quotient, and sex, were assessed using the Adaptive Behavior Assessment System—Second Edition, the Child Behavior Checklist, and the Childhood Autism Rating Scale—Second Edition.

**Results:**

Group comparisons revealed no significant differences in adaptive functioning or emotional-behavioral symptoms, with both groups showing marked adaptive deficits and borderline levels of social withdrawal. In contrast, clear differences emerged in autistic symptomatology, although certain items (e.g., imitation, adaptation to change, listening response, sensory response, and fear or nervousness) did not differ significantly.

**Discussion:**

These findings underscore the complexity of early differential diagnosis between ASD and GDD and emphasize the importance of enhanced clinician training and tailored early interventions to improve diagnostic accuracy and individualized care planning.

## 1 Introduction

Autism Spectrum Disorder (ASD) is a neurodevelopmental disorder characterized by deficits in social interaction and communication, restricted interest, and repetitive patterns of behavior (American Psychiatric Association, 2013). Symptoms typically emerge early in development and are usually recognized during the second year of life (Anagnostou et al., 2014; Bejarano-Martín et al., 2020; Fekar Gharamaleki et al., 2022). However, symptoms may be observed before 12 months if developmental delays are severe, or noted later than 24 months if the symptoms are more subtle (American Psychiatric Association, 2022). The clinical manifestations of ASD are heterogeneous, with considerable variability in both language and intellectual functioning (Munson et al., 2008; Tek et al., 2014). Early diagnosis during preschool years is essential for effective intervention (Lovaas and Smith, 2003; Coonrod and Stone, 2005; Tachibana et al., 2018; Charman, 2021). However, in preschool-aged children, some individuals with developmental delay may display traits characteristic of ASD without fully meeting the diagnostic criteria for ASD. The Diagnostic and Statistical Manual of Mental Disorders, Fifth Edition (DSM-5) (American Psychiatric Association, 2013) includes the diagnosis of Global Developmental Delay (GDD) to describe children under the age of five who present significant delays in at least two areas of neurodevelopmental, including gross or fine motor skills, speech/language, cognitive abilities, social/personal functioning, and activities of daily living (Shevell, 2008; Moeschler et al., 2014; Khan and Leventhal, 2025). As children with GDD grow, they are at increased risk of later being diagnosed with Intellectual Disability during school age (American Psychiatric Association, 2013). However, this is not always the case, highlighting the challenges of accurately assessing abilities in preschool-aged children, the potential for developmental trajectories to evolve over time (Lee et al., 2022) and the critical importance of early interventions (Dong et al., 2023).

The differential diagnosis between ASD and GDD is often challenging due to the frequent overlap and similarities between the two conditions. The heterogeneous clinical presentations and varied etiologies of ASD and GDD further complicate diagnostic differentiation, and comorbidity is relatively common (Coe et al., 2019; Miller et al., 2019; Shan et al., 2022). For instance, children with GDD may display limited early social-communication skills—such as reduced use of gestures and poor coordination of triadic gaze shifts between objects and people– making it difficult to distinguish GDD from ASD (Ventola et al., 2007; Veness et al., 2014).

Moreover, many children with GDD exhibit autism-like deficits, including atypical behavior and attentional difficulties (Einfeld and Tonge, 1996; Brereton et al., 2002; Gardner et al., 2018). Language delays and motor difficulties are also common to both conditions (American Psychiatric Association, 2013). While motor stereotypies (e.g., wiggling and flicking) are diagnostic criteria for ASD, they are not pathognomonic of the autistic spectrum; indeed, hand flapping is often observed in preschool-age children with GDD as well (Vig and Jedrysek, 1999). Shan et al. (2022) additionally found that children with more severe GDD presentations were more likely to exhibit ASD-like symptoms.

Adaptive functioning impairment is a common feature in both conditions (American Psychiatric Association, 2013; Shevell et al., 2007; Davico et al., 2022) with children with ASD and GDD showing adaptive skills below age expectations. Specifically, previous research has indicated that ASD is often associated with greater impairment within the social domain (Davico et al., 2022; Gabriels et al., 2007; Paul et al., 2014; Siracusano et al., 2021). Controlling for cognitive level, social impairment distinguishes children with ASD from those with other neurodevelopmental disorders (Mouga et al., 2015). The association between the severity of autistic symptoms and adaptive functioning, however, remains unclear. While some studies have reported an inverse relationship -indicating higher symptom severity correlates with lower adaptive performance (Szatmari et al., 2015; McDonald et al., 2015) others have found no significant link between the two variables (Ray-Subramanian et al., 2011; Yang et al., 2016; Hodge et al., 2021). Overall, more pronounced adaptive deficits have been observed when ASD co-occurs with GDD, particularly in the areas of social competences, personal autonomy, and daily living skills (Mouga et al., 2015; Liss et al., 2001; Perry et al., 2009; Green and Carter, 2014). In such cases, children tend to exhibit slower progress in social development compared to those with ASD alone or ASD combined with language impairment (Zachor et al., 2007; Bennett et al., 2014).

Early identification and accurate differential diagnosis are essential for guiding appropriate intervention strategies (Márquez-Caraveo et al., 2021). Although the clinical profiles associated with ASD and GDD share some common features, they entail distinct needs that should be addressed according to the individual's cognitive and adaptive profile (Ben-Itzchak and Zachor, 2007; Ben Itzchak et al., 2008). Recent evidence suggests that ASD intervention should primarily focus on enhancing social-communication skills and managing aberrant behavior, whereas GDD interventions typically emphasize structured daily routines, cognitive-behavioral therapy and other developmentally appropriate supports (Patel et al., 2020; Wickstrom et al., 2021).

Consistent with existing literature, differentiating and diagnosing ASD in preschool-aged children with developmental delays is often challenging due to the comorbidities and overlapping clinical manifestations. The present study therefore sought to examine differences and similarities in adaptive functioning, emotional/behavioral features, and autistic symptomatology in preschoolers formally diagnosed with ASD and those with GDD who exhibit autistic traits but do not meet full diagnostic criteria for ASD. Based on prior evidence, we hypothesized that both groups would present clinically significant emotional symptoms and marked adaptive impairments, with the ASD group demonstrating more pronounced social deficits and greater severity of autistic symptomatology. At the same time, we anticipated some shared symptomatology across groups, potentially complicating differential diagnosis. To the best of our knowledge, this is the first study to explore such differences in an Italian preschool sample of children with GDD presenting with autistic-like symptoms. The findings may be valuable for clinicians in supporting the diagnostic process and promoting effective and early interventions, both in the clinical management of children and in psychoeducational and preventive support for parents.

## 2 Materials and methods

### 2.1 Participants

This retrospective study included 89 children aged 3 to 5.8 years, recruited at the Child and Adolescents Neuropsychiatry Unit of the Hospital. The sample, matched for age, IQ and sex, was divided into two groups based on DSM-5 clinical diagnosis: 42 children diagnosed with ASD (ASD group) and 47 with GDD (GDD group). Demographic features are summarized in Table 1.

**Table 1:** Demographic characteristics of the sample.

	**ASD M (SD)**	**GDD M (SD)**	***P***
Age	4.01 (0.73)	3.75 (0.81)	0.111
IQ	65.79 (20.88)	69.66 (18.86)	0.36
Sex (M/F)	33/9	41/6	0.276

ASD, autism spectrum disorder; GDD, global developmental delay; IQ, intelligent quotient; M, mean; SD, standard deviation.

Inclusion criteria required that children were undergoing their first clinical evaluation and exhibited social-communication atypicalities and autistic traits, regardless of their final diagnosis. Exclusion criteria included children with isolated diagnoses of: (1) communication disorders, (2) movement disorders, (3) language delays, 4) GDD in the absence of autism-like features.

The study was conducted in accordance with the guidelines of the Declaration of Helsinki and was approved by the local Ethics Committee (practice number 3341/2024, protocol number 211, approval date: April 22, 2024).

### 2.2 Procedure

Diagnostic evaluations were conducted by a multidisciplinary team consisting of child psychiatrists, neuropsychologists, and speech therapists. The comprehensive assessment included developmental history, detailed clinical observations and standardized neuropsychological evaluations, covering cognitive, language, psychomotor, social-communication, psychopathological and adaptive functioning assessment. All assessments were carried out as part of routine clinical practice at the Child and Adolescents Neuropsychiatry Unit of the Hospital and were typically completed over two working days. All participants and parents were informed about assessment instruments and treatment options.

The clinical diagnosis of ASD was established according to DSM-5 criteria through multidisciplinary consensus, integrating developmental history, direct clinical observation, cognitive and adaptive assessments, and standardized instruments. The Childhood Autism Rating Scale, Second Edition (CARS-2) (Schopler et al., 2010) was used as a supportive measure to quantify autistic symptom severity, yet diagnostic classification relied on clinical judgment rather than on test cutoffs.

The diagnosis of GDD was likewise made according to DSM-5 criteria, with ASD explicitly ruled out despite the presence of autistic traits, as the full diagnostic criteria for ASD were not met. In both groups, diagnostic classification was based on team consensus and the integration of multiple sources of information. The CARS-2 contributed to characterizing autistic traits but not to confirming or excluding ASD diagnosis.

Diagnostic decisions were made by a single, stable multidisciplinary team composed of child psychiatrists, psychologists and speech therapists, all with extensive experience in neurodevelopmental disorders and long-term practice within the hospital's child and adolescent neuropsychiatry unit. Diagnostic conclusions were reached by integrating all available information from the assessments, and final classifications were established through team meetings and consensus discussions, ensuring consistency and reliability across cases.

### 2.3 Measures

#### 2.3.1 Cognitive assessment

Cognitive development was assessed through Griffiths Scales of Child Development – Third Edition (Griffiths III) (Green et al., 2016) and Leiter International Performance Scale – Third Edition (Leiter-3) (Roid and Miller, 2013). Griffiths III scales provide a direct measure of child psychomotor development from birth to 6 years old, assessing five developmental domains: *Foundations of Learning* (A Scale), *Language and Communication* (B Scale), *Eye and Hand Coordination* (C Scale), *Personal-Social–Emotional* (D Scale), and *Gross Motor Skills* (E Scale). An overall development score is also calculated. The raw scores of each scale and the overall developmental score are converted into Age Equivalent scores and Developmental Quotients.

*Leiter-3* is a non-verbal test designed to measure IQ and cognitive ability across a wide age range, from 3 to 80 years. The Leiter-3 test is entirely non-verbal, including instructions, which are conveyed through non-verbal means. It consists of four subtests administered in the following order: *Figure Ground*, where participants must identify a shape embedded within a complex figure; *Form Completion*, which requires participants to determine the form resulting from the combination of elements; *Classifications and Analogies*, where participants must classify forms and to reason through object matrices; and *Sequential Order*, which requires participants to discover the rule governing a series of forms and coherently add additional elements.

#### 2.3.2 Adaptive functioning

Adaptive functioning was assessed through Adaptive Behavior Assessment System - Second Edition (ABAS II) (Ferri et al., 2014). The ABAS II is a standardized questionnaire designed to evaluate adaptive skills in children from birth to 21 years of age. The instrument assesses ten adaptive areas grouped into three domains: conceptual (communication, preschool/school skills, self-control), social (play/leisure, socialization) and practical (self-care, home/school life, use of the environment, health and safety, work). Additionally, motor skills are evaluated for children aged 0 to 5 years. The instrument provides a comprehensive score known as the General Adaptive Composite (GAC). Normative data offer composite scores for GAC, and the conceptual (CAD), social (SAD) and practical (PAD) domains, with a mean (M) ± standard deviation (SD) normal range 100 ± 15. The nine subscales also have a mean ± standard deviation normal range 10 ± 3, which completes the questionnaire.

#### 2.3.3 Behavioral and psychopathological assessment

To assess the emotional and behavioral profile, the Child Behavior Checklist (CBCL) (Achenbach and Rescorla, 2001) was used. The CBCL is a standardized parent/caregiver-report questionnaire used to assess emotional, behavioral, and social problems in children. The preschool version (CBCL/1.5–5) is designed for children aged 1.5 to 5 years. The hierarchical structure of the CBCL includes 113 items and several scales (*Syndrome Scales, Internalizing, Externalizing, Total Problems Scales* and *DSM-Oriented Scales*). For this study, we focused on the *Syndrome Scales (*which included Emotionally Reactive, Anxious/Depressed, Somatic Complaints, Withdrawn, Sleep Problems, Attention Problems, and Aggressive Behavior) as these scales do not contain overlapping items.

According to the ASEBA Assessment Data Manager (ADM), for *Syndrome Scale Scores*, a t-score ≤ 64 indicates non-clinical symptoms (“non-clinical”), a t-score between 65 and 69 indicates that the child is at risk for problem behaviors (“borderline”), and a t-score ≥ 70 indicates clinical symptoms (“clinically-relevant”).

Although the CBCL is a broad-band instrument, it has shown good sensitivity in distinguishing children with ASD from both typically developing peers and those with other psychiatric disorders on specific scales, particularly Withdrawn and Pervasive Developmental Problems (Rescorla et al., 2015; Limberg et al., 2017; Chericoni et al., 2021).

#### 2.3.4 Autism assessment

To evaluate autistic symptoms, the Childhood Autism Rating Scale, Second Edition-Standard Version (CARS2-ST) (Schopler et al., 2010) was used. The CARS is a widely used clinical tool for identifying autism and assessing the severity of ASD symptoms (Schopler et al., 1980). In this study, we employed the CARS2-ST, which is recommended for use with children under 6 years of age, or with older children presenting with an IQ ≤ 79. The scale consists of 15 items, each rated by trained clinicians based on a combination of direct observation and structured interviews with the primary caregiver. Each item is scored on a 4-point scale, ranging from 1 (age-appropriate behavior) to 4 (severely atypical behavior for age). The items address the following functional areas: *Relating to People, Imitation, Emotional Response, Body Use, Object Use, Adaptation to Change, Visual Response, Listening Response, Taste Smell and Touch Response and Use, Fear or Nervousness, Verbal Communication, Non-verbal Communication, Activity Level, Level and Consistency of Intellectual Response, General Impressions*. The total score is calculated by summing the individual item scores, resulting in a possible range from 15 to 60. A total score of < 30 suggests the absence of ASD, 30–36.5 indicates mild to moderate ASD symptoms, and a total score of ≥37 indicates severe ASD. Reliability and validity studies of the CARS-2 have demonstrated strong agreement with standardized diagnostic instruments, such as the Autism Diagnostic Observation Schedule, Second Edition (ADOS-2) (Ji et al., 2023), as well as high concordance with clinical diagnosed based on the DSM-5 criteria (American Psychiatric Association, 2013; Mayes et al., 2014; Dawkins et al., 2016).

#### 2.3.5 Data analysis

Descriptive statistics were used to analyze demographic and clinical characteristics of the whole sample. Chi-squared test was used to determine differences in categorical variables. Group differences were examined by *t* test and analysis of covariance (ANCOVA) with age, sex, and IQ as covariates. Partial eta squared (ηp2) was used to measure effect size. A *p*-value less than or equal to 0.05 was considered as statistically significant. To account for multiple comparisons, a Benjamini-Hochberg correction for false discovery rate was applied to adjust the *p*-values.

## 3 Results

### 3.1 Adaptive functioning

The analysis of ABAS II scores failed to detect significant differences between groups. Table 2 summarizes results.

**Table 2:** Differences between ASD and GDD groups at ABAS II scores.

**ABAS II domains**	**ASD M (SD)**	**GDD M (SD)**	***P***	**ηp^2^**
Conceptual domain	68.48 (17.39)	71.96 (20.3)	0.887	< 0.01
Social domain	68.88 (14.13)	73.32 (17.64)	0.456	0.006
Practical domain	71.79 (17.71)	71.39 (17.51)	0.472	0.006

ASD, autism spectrum disorder; GDD, global developmental delay.

### 3.2 Psychopathological and behavioral symptoms

No significant differences emerged between groups for any of the considered Child Behavior Checklist scores. The results are summarized in Table 3.

**Table 3:** Differences between ASD and GDD groups at Child Behavior Checklist scores.

**CBCL scales**	**ASD M (SD)**	**GDD M (SD)**	***P***	**ηp^2^**
Emotionally reactive	59.43 (7.91)	58.1 (11.5)	0.460	0.007
Anxious/depressed	57.57 (8.78)	58.91 (8.95)	0.681	0.002
Somatic complaints	56.05 (7)	56.66 (8.78)	0.807	0.001
Withdrawn	66.29 (7.4)	66.87 (11.83)	0.738	0.001
Sleep problems	53.69 (5.92)	57.51 (11.06)	0.101	0.032
Attention problems	60.38 (8.67)	61.96 (8.67)	0.584	0.004
Aggressive behavior	56.17 (7.71)	56.87 (11)	0.938	< 0.001

ASD, autism spectrum disorder; GDD, global developmental delay; IQ, intelligent quotient; M, mean; SD, standard deviation.

### 3.3 Autistic symptoms

As expected, children in the ASD group exhibited significantly higher scores in the majority of CARS-2 items; of note, these results resisted after the correction for multiple comparisons (Benjamini-Hochberg method). However, for some items—namely Imitation, Adaptation to Change, Listening Response, Taste, Smell, and Touch Response and Use, Fear or Nervousness, Level and Consistency of Intellectual Response—the two groups exhibited similar scores. Table 4 summarize the results.

**Table 4:** Differences between ASD and GDD groups at CARS-2 scores.

**CARS-2 items**	**ASD M(SD)**	**GDD M(SD)**	***P***	**ηp^2^**
Relating to people	2.68 (0.48)	2.15 (0.79)	< 0.001^***∧^	0.144
Imitation	2.19 (0.74)	1.89 (0.73)	0.166	0.023
Emotional response	2.45 (0.52)	2.1 (0.54)	0.007^**∧^	0.085
Body use	2.33 (0.57)	1.99 (0.61)	0.024^*∧^	0.06
Object use	2.48 (0.54)	1.99 (0.64)	< 0.001^***∧^	0.148
Adaptation to change	2.12 (0.72)	1.98 (0.7)	0.249	0.016
Visual response	2.34 (0.47)	1.84 (0.52)	< 0.001^***∧^	0.215
Listening response	2.05 (0.66)	1.86 (0.6)	0.286	0.014
Taste, smell, and touch response and use	1.76 (0.65)	1.58 (0.57)	0.192	0.02
Fear or nervousness	2.11 (0.63)	1.97 (0.7)	0.312	0.012
Verbal communication	2.96 (0.47)	2.63 (0.54)	0.001^**∧^	0.117
Nonverbal communication	2.56 (0.59)	2.12 (0.64)	0.003^**∧^	0.099
Activity level	2.24 (0.5)	1.82 (0.55)	< 0.001^***∧^	0.144
Level and consistency of intellectual response	1.86 (0.66)	1.8 (0.66)	0.818	0.001
General impressions	2.53 (0.53)	1.95 (0.59)	< 0.001^***∧^	0.232

ASD, autism spectrum disorder; GDD, global developmental delay; IQ, intelligent quotient; M, mean; SD, standard deviation.

^*^p < 0.05.

^**^p < 0.01.

^***^p < 0.001.

^***∧^, resisting after controlling for multiple comparisons by applying Benjamini-Hochberg method.

## 4 Discussion

The aim of the present study was to examine differences and similarities in the clinical profiles of preschoolers formally diagnosed with ASD and those with GDD who nonetheless display autistic traits without meeting full diagnostic criteria. Specifically, we investigated potential differences in adaptive functioning and emotional/behavioral profiles. We also analyzed autistic symptomatology, with particular attention to identifying the most salient features that may discriminate between the two groups, as well as shared characteristics that make differentiation more challenging.

With regard to adaptive functioning, no significant differences emerged between preschoolers with ASD and those with GDD based on parent reports. In both diagnostic groups, children exhibited marked impairments across all adaptive domains -conceptual, social, and practical- with a similar distribution of deficits. This finding is consistent with the existing literature indicating that adaptive deficits are common across neurodevelopmental (American Psychiatric Association, 2013) and are not necessarily linked to intellectual functioning (Mathiassen et al., 2012; Lindblad et al., 2013). Although prior research in ASD has often highlighted greater social and daily living difficulties compared to other conditions (Gabriels et al., 2007; Paul et al., 2014; Mouga et al., 2015; Chatham et al., 2018), the present results instead support a relatively homogeneous impairment profile, as reported by other studies (Davico et al., 2022; Siracusano et al., 2021; Hamner et al., 2019). Given that the mean cognitive level of the ASD sample was below the normative range, it is plausible that adaptive functioning was further compromised compared to individuals with ASD and normative cognitive abilities (Hodge et al., 2021; Perry et al., 2009; Green and Carter, 2014). Nevertheless, the discrepancy between cognitive abilities and adaptive functioning remains significant even in higher-functioning individuals with ASD (Alvares et al., 2020). Overall, the presence of overt autistic symptomology in ASD did not appear to affect adaptive functioning differently form the subclinical autistic traits observed in GDD, consistent with studies finding no clear link between symptom severity and adaptive deficits (Yang et al., 2016; Hodge et al., 2021).

Regarding emotional and behavioral functioning, no significant differences were identified between the two groups, indicating a broadly similar profile. In both the ASD and GDD groups, mean scores for emotional and behavioral problems generally fell within the non-clinical range, with the sole exception of the Withdrawn scale of the CBCL, on which both groups obtained borderline mean scores. This subscale reflects internalizing difficulties such as a preference for solitude, limited interest in social interactions, avoidance of contact, lack of communicative initiative, and apparent sadness (Achenbach and Rescorla, 2001). Although these features are frequently associated with ASD, as confirmed by numerous studies reporting elevated scores on this scale in children with the condition (Rescorla et al., 2015; Limberg et al., 2017; Chericoni et al., 2021; Sikora et al., 2008; Muratori et al., 2011; Havdahl et al., 2016; Rescorla et al., 2020), they are not exclusively linked to this diagnosis. Other research has shown that children with other developmental challenges may also obtain scores in the clinical range on this scale (Rescorla et al., 2015; Muratori et al., 2011; Havdahl et al., 2016; So et al., 2013; Ooi et al., 2011; Myers et al., 2014). Consistent with this body of evidence, our results showed that both groups exhibited comparable levels of social withdrawal and reduced engagement. These shared behavioral characteristics have important implications for intervention, underscoring the need to promote strategies aimed at reducing these difficulties, while also highlighting how such overlapping behavioral features can complicate the differential diagnosis between ASD and GDD.

Regarding autistic symptomatology, as expected, statistically significant differences emerged between the two groups, with the ASD group showing higher scores on most CARS items (Relating to People, Emotional Response, Body and Object Use, Visual Response, Verbal and Non-Verbal Communication, Activity Level, and General Impression). Interestingly, some items did not differ significantly between groups, suggesting overlapping clinical presentations in specific domains. For example, *Imitation* item scores were mildly atypical in both groups, despite imitation deficits being commonly linked to ASD (Williams et al., 2004; Vivanti and Hamilton, 2014). In this context, Vivanti and Hamilton (2014) demonstrated that imitation comprises two components—accuracy and propensity to imitate—and found that while both ASD and GDD groups were similarly impaired in accuracy (likely influenced by cognitive functioning), they differed in propensity, which is more closely linked to social motivation. Therefore, the comparable CARS imitation scores in our sample may reflect shared deficits in accuracy, with the scale's unitary assessment unable to capture differences in propensity. Unsurprisingly, no group difference emerged on the *Intellectual Response* item, consistent with the comparable cognitive functioning observed across groups.

Another item for which group differences did not emerge was *Adaptation to Change*, which assesses difficulties in modifying established habits, routines, or behavioral patterns, as well as challenges in transitioning from one activity to another (Schopler et al., 2010). While behavioral rigidity and insistence on sameness are hallmark features of ASD (Eisenberg et al., 2015), similar tendencies are also commonly observed in children with GDD, who may display behaviors such as a preference for familiar routines and daily patterns (Mooney et al., 2009), serving as compensatory strategies to cope with deficits in planning, cognitive flexibility, and behavioral organization. In contrast, in ASD, these behaviors appear linked to regulation of emotional arousal and anxiety (Leekam et al., 2011; Samson et al., 2015; Baribeau et al., 2023; Rodríguez-Jiménez and Martínez-González, 2024). Overall, difficulties in adapting to change and reliance on structured routines are common to both ASD and GDD, but likely serve distinct functions, with important implications for interventions. Implementing consistent daily routines has been associated with reductions in behavioral problems among children with neurodevelopmental delays (Hatherly et al., 2023), whereas targeted behavioral interventions in ASD are crucial to reduce repetitive behaviors that interfere with daily functioning and emotional self-regulation (Sevin et al., 2015).

Similarly, scores on the *Taste, Smell, and Touch Response and Use* and the *Listening Response* items did not significantly differ between the ASD and GDD groups. According to the CARS manual (Schopler et al., 2010), these items assess responsiveness to proximal sensory modalities (gustatory, olfactory, and tactile stimuli, including pain) and “distal” senses (hearing), respectively. These domains represent distinct components of sensory processing, yet the similarity in scores between groups aligns with evidence that atypical sensory profiles, while characteristic of ASD, are not exclusively to this condition. Indeed, children with ASD frequently show hypo- or hyper-reactivity to both proximal (Ben-Sasson et al., 2009; Baranek et al., 2013, 2018) and distal stimuli (Chang et al., 2015). However, similar alterations have been reported in children with neurodevelopmental delays, particularly in the presence of cognitive impairment, suggesting that sensory modulation difficulties may represent a transdiagnostic feature across neurodevelopmental conditions (Baranek et al., 2006; McCormick et al., 2016; Cardon, 2018; Ringold et al., 2022). Notably, among all sensory modalities, visual processing emerged in our data as the one most clearly distinguishing ASD from GDD. This finding supports the notion that atypical visual responses may constitute a specific marker of ASD, as indicated in the DSM-5 criteria (American Psychiatric Association, 2013), and may help differentiated children with autistic traits from those with a formal ASD diagnosis.

No significant group differences emerged on the *Fear or Nervousness* item, which assesses the presence of unusual or inappropriate fears, as well as the absence of fear in contexts that would typically evoke anxiety in typically developing peers. This finding may reflect shared difficulties in environmental reactivity and emotional regulation, domains that have been well-documented in ASD (Samson et al., 2015; Berkovits et al., 2017) but are also increasingly recognized in other developmental disorders (England-Mason, 2020), including GDD (Davico et al., 2022). It is therefore plausible to consider that impairments in the ability to regulate emotions, particularly in response to external stimuli, may contribute to similar clinical manifestation related to fear and nervousness in both children with ASD and those with GDD (Davico et al., 2022).

Considering the evidence presented, our findings highlight the considerable difficulty in distinguishing, during the preschool years, between children who exhibit autistic traits but do not meet full diagnostic criteria and those with a clear diagnosis of ASD, with important implications for both assessment and intervention. First, clinicians require specialized training in the assessment of preschool-aged children, with a focus on understanding comorbidities and accurately differentiating among neurodevelopmental disorders. Such training may minimize the risk of misdiagnosis and ensures proper recognition of the distinctive features of specific diagnostic profiles. A comprehensive understanding of each child's profile allows for the design of tailored therapeutic interventions that address multiple development while actively involving parents. Supporting caregivers in understanding and managing their child's specific challenges may reduce parental stress, improve overall family functioning, and foster a more stable and responsive environment for the child's development.

Although this study holds significant implications for improving the accurate diagnostic differentiation of preschool children with and without an autism diagnosis, several limitations must be acknowledged. First, the relatively small sample size constrains the generalizability of the findings. Second, the study included only clinically referred children without a comparison group of typically developing peers, further restricting the applicability of the results. Third, the assessment of adaptive functioning and emotional symptomatology relied on parent-report questionnaires, which may not fully capture key factors influencing how parents perceive and report their child's behaviors. Such factors may include parental stress, parental psychiatric conditions, family history of neurodevelopmental disorders, and socio-economic status. Future research should account for these variables to better understand their impact on children's functioning and diagnosis. Moreover, the CARS-2 scale, while used to quantify autistic symptomatology in both groups, also contributed as a supportive element during the diagnostic evaluation. Therefore, group differences on this measure should be interpreted with caution, as they may partially reflect aspects of the diagnostic process rather than fully independent group differences.

Despite these limitations, the present study has important clinical and therapeutic implications. It highlights the complexity of differential diagnosis in early childhood, particularly in cases where overlapping characteristics make distinctions between conditions challenging. In light of these challenges, our findings point to two key priorities: first, enhancing clinician training in the assessment of preschool-aged children to improve diagnostic accuracy across neurodevelopmental disorders; second, developing tailored therapeutic programs that actively involve parents and support them in understanding and managing their child's developmental difficulties. Strengthening both professional expertise and parental support may facilitate earlier identification and ultimately improve children's developmental outcomes.

## Data Availability

The raw data supporting the conclusions of this article will be made available by the authors, without undue reservation.
